# Delta-8 gummies causing visual snow: a case report

**DOI:** 10.3389/fopht.2023.1349525

**Published:** 2024-01-29

**Authors:** Michael S. Vaphiades

**Affiliations:** Department of Ophthalmology, University of Alabama, Birmingham, AL, United States

**Keywords:** visual snow (VS), visual snow syndrome (VSS), delta-8 gummies, delta-8 tetrahydrocannabinol, hallucinogen persisting perception disorder (HPPD)

## Abstract

A 26-year-old man developed visual snow syndrome (VSS) after consuming a little less than half of a delta-8 gummy (estimated at 4 mg of delta-8 tetrahydrocannabinol). Secondary VSS and hallucinogen-persisting perception disorder (HPPD) are discussed, and clinicians who evaluate patients with VS and VSS should ask about delta-8 gummies as an etiology of secondary VSS.

## Case

A 26-year-old man developed visual snow (VS), palinopsia, and photopsias the morning after consuming a little less than half of a delta-8 gummy, each of which contains 10 mg of delta-8 tetrahydrocannabinol (estimated at 4 mg). He had never taken one before and no other substances were consumed. The next morning, he experienced VS, which has persisted for the last year and continues to be an annoyance. Given the associated palinopsia and photopsia he experienced, his symptoms fulfill the diagnostic criteria of visual snow syndrome (VSS) (1). There was no history of tinnitus or migraine headaches. He did not smoke cigarettes or marijuana or drink alcohol and had never used illicit drugs. He had no medical history and did not take any medications. He arrived with a normal brain MRI in hand.

Neuro-ophthalmologic examination showed a blood pressure of 113/68 mmHg and a weight of 195 pounds. Visual acuity was 20/20 in both eyes (OU) and his color vision was 8/8 OU according to the Ishihara pseudo-isochromatic color plate test. Automated perimetry and confrontation fields were normal OU. His pupils measured 8 mm OU and were normally reactive without a relative afferent pupillary defect. Ocular motility and slit lamp examinations were normal. Ophthalmoscopy was normal OU. Optical coherence tomography of the macula was normal.

## Discussion

The phenomenon of VS was first described by Liu et al. in 1995 as an “unusual complication of migraine” manifesting as “persistent diffuse small particles such as TV static, snow, lines of ants, dots, and rain” occupying the patient’s entire visual field (2). VS is a continuous pan-field visual disturbance (usually black and white or transparent dots) similar to looking at an old analog television with poor reception (1, 3). This disorder was first referred to as a “phenomena” in 2005 (4) and a “syndrome” in 2014 (1) and later more commonly as VSS (5) with VS as the defining characteristic in addition to other visual and perceptual symptoms (1). In 2014, Schankin et al. proposed a definition of VSS as dynamic continuous tiny dots across the entire visual field, persisting for 3 months plus at least two of the following four elements: 1) palinopsia; 2) enhanced entoptic phenomena (with at least one of the following symptoms: excessive floaters in both eyes, excessive blue field entopic phenomena [uncontrollable grey/white/black dots or rings shooting over the visual field in both eyes when looking at homogeneous bright surfaces such as the sky), self-light of the eye [colored waves or clouds when closing the eyes in the dark], or spontaneous photopsia); 3) photophobia; and 4) nyctalopia, with symptoms not consistent with typical migraine visual aura and not better accounted for by another disorder (1). In 2018, these criteria were adopted by the International Headache Society (6). Associated symptoms that may occur with VSS include migraine (reported in approximately 50%–80% of cases) (7–10), concentration problems (80%), tinnitus (approximately 50%–60%), irritability (55%), and lethargy (40%) (10). However, a recent web-based survey of 1,100 patients with VSS showed that tinnitus had the highest prevalence and migraine was second, both of which were closely associated with the severity of the VSS, which should be viewed as a spectrum from mild to severe (3).

It is important to divide VSS into “primary” and “secondary VSS.” Primary VSS is defined as symptoms that develop without an inciting event or known cause, and secondary VSS is defined as symptoms arising from a secondary cause such as head trauma (immediate or delayed onset), hallucinogenic drugs, or other chronic neurological or ophthalmologic disorders (7).

Hallucinogen-persisting perception disorder (HPPD) is characterized by the re-emergence of perceptual symptoms during acute hallucinogen intoxication after cessation of use (11). It occurs from using “classic hallucinogens”, such as lysergic acid diethylamide (LSD) (12); however, cannabinoids have also been included in the development of the disorder (11–13). Ford et al. retrospectively reviewed 13 clinic patients with HPPD and 24 case reports in the literature. The most frequent symptoms were visual snow, floaters, palinopsia, photophobia, and nyctalopia. The majority of patients had ongoing symptoms. They found that symptoms of HPPD overlap with the typical features of VSS. They concluded that patients presenting with VSS should be screened for past recreational drug use and that the DSM-5 description of HPPD does not include visual snow, nyctalopia, photophobia, or floaters. In this study, LSD, 3,4-Methyl enedioxy methamphetamine (MDMA), and cannabinoid use were common (11).

Delta-8 tetrahydrocannabinol (delta-8 THC) combined with fruit-flavored candy is called a delta-8 gummy. These very popular substances are widely available, advertised on television, and not regulated by the United States Food and Drug Administration.

The obvious limitation of this report is that there is only one case, thus I cannot generate statistical information on the incidence or prevalence of VS and VSS in this patient population. However, this single case may facilitate a larger case series in which we may make more meaningful correlations between the population in question that consumes delta-8 gummies and their medical history of other positive visual phenomena, including phosphenes, photopsias, complex hallucinations, palinopsia, migraine, and tinnitus. This larger series may even include genetic data because there may be a genetic link that renders certain individuals vulnerable to VSS triggers, such as delta-8 gummies.

In our patient, the temporal relationship of the onset of VSS to the consumption of delta-8 gummies is compelling. It highlights that in this particular individual, only 4 mg of delta-8 tetrahydrocannabinol (approximately less than half a gummy) was enough to cause persistent VSS. One implication of this case report is that it may give pause to individuals who have never consumed this product because of the unwanted side effect of VSS. In addition, the case may cultivate interest in a larger study as mentioned in the limitations. For clinicians who evaluate patients with VS and VSS, it seems prudent to ask about delta-8 gummies as an etiology of secondary VSS.

## Author contributions

MV: Conceptualization, Writing – original draft.
